# The expression of Nectin-4 on the surface of ovarian cancer cells alters their ability to adhere, migrate, aggregate, and proliferate

**DOI:** 10.18632/oncotarget.14206

**Published:** 2016-12-25

**Authors:** Kristin L.M. Boylan, Petra C. Buchanan, Rory D. Manion, Dip M. Shukla, Kelly Braumberger, Cody Bruggemeyer, Amy P.N. Skubitz

**Affiliations:** ^1^ Department of Laboratory Medicine and Pathology, University of Minnesota, Minneapolis, MN, USA

**Keywords:** Nectin-4, ovarian cancer, cell adhesion, spheroids, migration

## Abstract

The cell adhesion molecule Nectin-4 is overexpressed in epithelial cancers, including ovarian cancer. The objective of this study was to determine the biological significance of Nectin-4 in the adhesion, aggregation, migration, and proliferation of ovarian cancer cells. Nectin-4 and its binding partner Nectin-1 were detected in patients’ primary tumors, omental metastases, and ascites cells. The human cell lines NIH:OVCAR5 and CAOV3 were genetically modified to alter Nectin-4 expression. Cells that overexpressed Nectin-4 adhered to Nectin-1 in a concentration and time-dependent manner, and adhesion was inhibited by antibodies to Nectin-4 and Nectin-1, as well as synthetic Nectin peptides. In functional assays, CAOV3 cells with Nectin-4 knock-down were unable to form spheroids and migrated more slowly than CAOV3 parental cells expressing Nectin-4. NIH:OVCAR5 parental cells proliferated more rapidly, migrated faster, and formed larger spheroids than either the Nectin-4 knock-down or over-expressing cells. Parental cell lines expressed higher levels of epithelial markers and lower levels of mesenchymal markers compared to Nectin-4 knock-down cells, suggesting a role for Nectin-4 in epithelial-mesenchymal transition. Our results demonstrate that Nectin-4 promotes cell-cell adhesion, migration, and proliferation. Understanding the biology of Nectin-4 in ovarian cancer progression is critical to facilitate its development as a novel therapeutic target.

## INTRODUCTION

Ovarian cancer is the most lethal gynecological malignancy, resulting in over 14,000 deaths annually in the U.S. [1]. Due to the vague symptoms and lack of a screening test suitable for the general population, most women are diagnosed at a late stage of disease, when patients have a poor prognosis. Although most ovarian cancer patients will respond to initial treatment with surgery and chemotherapy, the majority relapse with chemoresistant disease [2].

The primary mechanism of ovarian cancer progression is by localized tumor shedding and seeding within the peritoneal cavity, although hematogenous metastasis has been observed [3]. Cells are released from the primary tumor, which may arise in the fimbria of the fallopian tube [4], and then seed throughout the peritoneal cavity, attaching to the local organs and invading, ultimately resulting in the death of the patient. Ovarian cancer cells are unique in their ability to recruit fluid into the peritoneal cavity (ascites fluid) and exist in a free-floating form as single cells and multicellular aggregates, termed spheroids. Spheroids are able to resist standard chemotherapy (which relies upon rapidly dividing cells), due to their slow replication and the protection afforded by the tight cell aggregation [5, 6]. Ovarian cancer spheroids are also capable of adhering to extracellular matrix proteins and monolayers of mesothelial cells [7–11] and can disaggregate and invade the peritoneal organs [8, 12–16].

We discovered the overexpression of the cell adhesion molecule Nectin-4 (PVRL4) in ovarian cancer tissues by gene microarray analysis [17]. Subsequently, we showed that Nectin-4 RNA and protein are overexpressed in ovarian cancer tissues and cell lines compared to their normal ovarian counterparts [18]. More recently, in a study of 25 ovarian cancer tumors, Nabih et al. [19] found that expression of Nectin-4 mRNA was increased in 97.4% of the ovarian cancer samples. Normal tissue expression of Nectin-4 is largely limited to the placenta; however, lower levels of expression are found in the skin, stomach, prostate, lung and trachea [20, 21]. We also showed that the cleaved, soluble extracellular domain of Nectin-4 (sN4) is detectable at elevated levels in the sera of ovarian cancer patients [18].

Nectin-4 overexpression has been reported in ductal breast carcinoma, lung adenocarcinoma, and pancreatic cancer; and high Nectin-4 expression in those tumors was associated with disease progression or poor prognosis [22–26]. Several of these studies also detected elevated levels of sN4 in cancer patients’ serum and, in the case of breast cancer, a correlation was shown between high levels of sN4 and number of metastases [24, 26]. A recent study demonstrated that an antibody-drug conjugate targeting Nectin-4 inhibited tumor growth in xenografts models of breast, bladder, pancreatic and lung cancer [27]. Clinical trials are underway to target carcinomas that express Nectin-4 by use of this drug-linked monoclonal antibody (mAb) against Nectin-4 [28].

Nectins are a family of four Ca^2+^-independent, immunoglobulin-like cell adhesion molecules important in the formation and maintenance of adherens junctions and tight junctions [29–33]. All nectins share a similar structure: three immunoglobulin-like extracellular loops, a single transmembrane region, and a short cytoplasmic domain that binds to afadin, through which nectins are connected to the actin cytoskeleton [21, 34–36]. The extracellular domains of nectins can be proteolytically cleaved to release a soluble fragment (sN4) which may regulate cell function [25, 37–39].

Nectins function as cell adhesion molecules by first forming homo cis-dimers on the cell surface and then trans-dimers on adjacent cells in both a homophilic and heterophilic manner. The specificity of binding is different for each nectin; Nectin-4 binds to itself and Nectin-1 (PVRL1) [21, 36, 40]. Cell-cell contacts are thought to be initiated by an interaction between nectins on adjacent cells. Subsequently, the cadherin-catenin complex is recruited to sites of nectin-based intercellular adhesion and the trans-interaction of cadherins on adjacent cells occurs, forming the adherens junction [29, 41].

The extracellular domains of some members of the nectin family bind to growth factor receptors, such as fibroblast growth factor receptor, platelet-derived growth factor receptor, or epidermal growth factor receptor (ERBB3 /HER3), which may play a role in the regulation of nectin function in cell proliferation, migration and apoptosis [42–49]. However, the role of Nectin-4 in cellular functions, beyond cell-cell adhesion, is not well understood. A recent study by Pavlova et al. [50] provided evidence that Nectin-4 promotes anchorage independence in breast cancer cells.

In this study, we examined the role that Nectin-4 plays in several cellular functions that underlie ovarian cancer progression: cell adhesion, spheroid formation, migration, and proliferation. These experiments offer insight into how ovarian cancer cells may act *in vivo* and may provide a rationale for the use of agents that target Nectin-4 in clinical trials.

## RESULTS

### Nectin-4 and its binding partner Nectin-1 are expressed in human mesothelial cells and ovarian cancer patient samples

**Figure 1:**
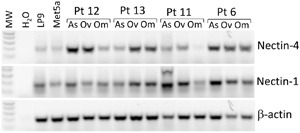
Nectin-4 and Nectin-1 are expressed in human mesothelial cells and ovarian cancer patient samples RT-PCR analysis of Nectin-4 and Nectin-1 expression in human mesothelial cell lines LP9 and Met5a, and matched samples from four patients with high grade serous ovarian cancer: ascites cells (As), primary ovarian tumor (Ov), and omental metastases (Om). Nectin-4 RNA was expressed in all of the samples, at variable levels. Nectin-1 RNA was more consistently expressed across samples. β-actin, loading control.

To assess the clinical relevance of the cell adhesion molecule Nectin-4 and its binding partner Nectin-1 in ovarian cancer, we examined their RNA expression in patient samples, as well as the human mesothelial cell lines LP9 and Met5a. RT-PCR analysis of matched ascites cells (As), primary ovarian tumor (Ov), and omental metastases (Om) from four patients with high-grade serous ovarian cancer showed that Nectin-1 was expressed in all samples tested in varying amounts (Figure 1). Nectin-4 was also expressed in all samples, although weakly in the omental sample from one patient. Furthermore, both Nectins were expressed in the mesothelial cell lines LP9 and Met5a (Figure 1). These data indicate that adhesion between Nectin-4 and Nectin-1 could contribute to ovarian cancer progression, and consequently may be a target for therapy.

### Generation and characterization of cell lines

**Figure 2:**
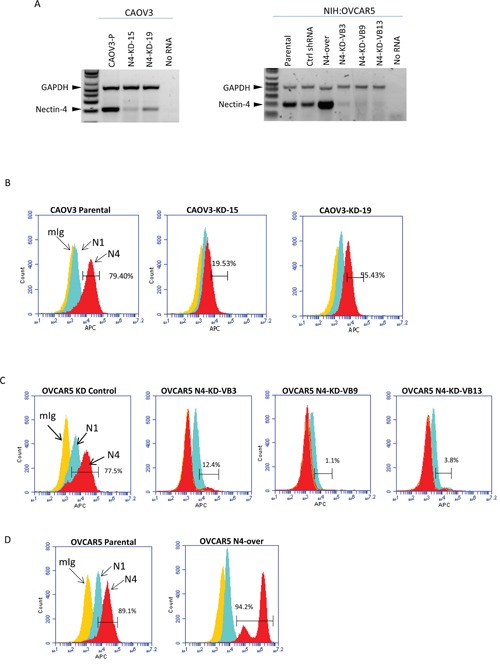
Characterization of CAOV3 and NIH:OVCAR5 cells for expression of Nectin-4 and Nectin-1 **A.** Cell lines were analyzed by RT-PCR for expression levels of Nectin-4 relative to the GAPDH control. **B-D.** Cells were analyzed by flow cytometry for the expression of Nectin-4 (N4, red), Nectin-1 (N1, light blue) or mouse IgG negative control (mIg, yellow). The percentage of cells that stained positively for Nectin-4 is shown in brackets. Cells tested were: (B) CAOV3 parental cells; single cell clones of CAOV3 stably expressing shRNA targeting Nectin-4 (CAOV3 N4-KD-15, and CAOV3 N4-KD-19). (C) NIH:OVCAR5 stably expressing control shRNA; clones of NIH:OVCAR5 cells stably expressing Nectin-4 shRNA (N4-KD-VB3, N4-KD-VB9, and N4-KD-VB13). (D) NIH:OVCAR5 parental cells; NIH:OVCAR5 over-expressing Nectin-4 (N4-over).

The human ovarian cancer cell lines CAOV3 and NIH:OVCAR5 were selected for this study in order to understand the potential function of Nectin-4 in ovarian cancer progression. These cell lines both express moderate levels of Nectin-4, relative to a dozen other human ovarian cancer cell lines that we had previously characterized [18], and thus are ideal for generating cell lines that have Nectin-4 expression knocked down. We created Nectin-4 knock-down cell lines by stable expression of a Nectin-4 targeting shRNA. Cells were transfected with lentivirus containing an shRNA sequence targeting Nectin-4 or control shRNA, and selected with puromycin. Clones of Nectin-4 shRNA expressing cells were screened by RT-PCR (reverse transcription – polymerase chain reaction) for reduced levels of Nectin-4 expression (Figure 2A), which was verified by flow cytometry (Figure 2B-2C). Two Nectin-4 shRNA clones were selected for CAOV3 (N4-KD-15 and N4-KD-19), which have Nectin-4 protein expression reduced by 75% and 30% relative to the parental cells, as determined by flow cytometry (Figure 2B, red histogram). All three selected Nectin-4 shRNA clones from NIH:OVCAR5 (N4-KD-VB3, N4-KD-VB9, and N4-KD-VB13) showed very little expression of Nectin-4 protein on the cell surface [87-99% Nectin-4 knock-down (Figure 2C, red histogram)] relative to the parental cell line (Figure 2D, red histogram) or cells that express the control shRNA. Both NIH:OVCAR5 and CAOV3 cells also express Nectin-1 (Figure 2B-2D, light blue histogram), which has been shown to serve as a binding partner for Nectin-4 [21]. The level of Nectin-1 expression in the engineered cells remained similar to that observed in the parental cell lines.

We used FACS (fluorescence activated cell sorting) to select NIH:OVCAR5 cells that overexpressed full-length Nectin-4 relative to the parental cell line (termed NIH:OVCAR5-N4-over) in order to facilitate *in vitro* adhesion assays. By flow cytometry, 94% of the NIH:OVCAR5-N4-over cells expressed Nectin-4 on the cell surface, compared to 89% of the parental NIH:OVCAR5 cells (Figure 2D), and approximately 75% of the NIH:OVCAR5-N4-over cells expressed a significantly higher number of Nectin-4 molecules per cell than the parental cells.

### Nectin-4 expression increases cell adhesion

**Figure 3:**
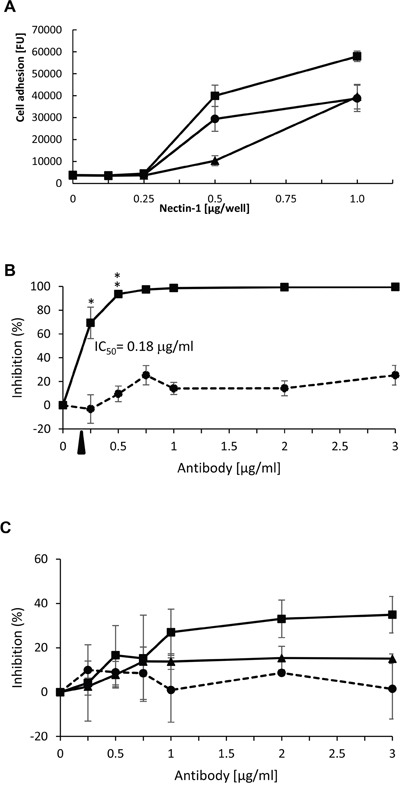
Adhesion of NIH:OVCAR5-N4-over ovarian cancer cells to Nectin-1 **A.**xs Microtiter plates were coated with increasing amounts of recombinant human Nectin-1 extracellular domain (R&D Systems). NIH:OVCAR5-N4-over cells were stained with CMFDA and incubated in the plate for 15 min (triangle), 30 min (circle), or 60 min (square). Adherent cells were quantified in a fluorescent plate reader. FU = fluorescent units; Error bars = SD. **B.** Microtiter plates were coated with 0.25 μg/well of recombinant human Nectin-1 extracellular domain (Acrobiosystems). NIH:OVCAR5-N4-over cells were pre-incubated for 30 min with increasing concentrations of mouse IgG (dashed line) or a mAb against Nectin-4 (solid line, mAb 2659); and then cells were incubated in the wells for 30 min. Inhibition of cell adhesion was calculated relative to the adhesion observed with no IgG added; the FU of BSA-coated control wells was subtracted from all samples. Shown is a representative of three independent experiments. Error bars = SD [%]. The IC50 of Nectin-4 mAb 2659 was calculated to be 0.18 μg/ml Student's t-test unpaired. ** = P<0.01, *=P<0.05. **C.** In parallel studies, plates coated with 0.25 μg/well of recombinant human Nectin-1 extracellular domain (Acrobiosystems) were incubated with increasing concentrations of mAbs against Nectin-1 (square, mAb 2880; triangle, MABT61) or mouse IgG (dashed line) for 30 min, and then cells were incubated in the wells for 30 min. Inhibition of cell adhesion was calculated relative to the adhesion observed with no IgG added; the FU of BSA-coated control wells was subtracted from all samples. Shown is a representative of three independent experiments. Error bars = SD [%].

NIH:OVCAR5-N4-over cells were tested for their ability to adhere to the recombinant extracellular domain of Nectin-1 and Nectin-4 in an *in vitro* binding assay. The cells adhered to increasing concentrations of recombinant Nectin-1 in a time-dependent manner (Figure 3A). However, although Nectin-4 has been reported to bind to itself in a homotypic manner [51], we were unable to detect adhesion of the NIH:OVCAR5-N4-over cells to recombinant Nectin-4 when cells were incubated for up to 1 h in wells coated with 1 μg of Nectin-4 per well. When NIH:OVCAR5 parental cells were tested in parallel studies, the level of adhesion to Nectin-4 and Nectin-1 was negligible, even when cells were incubated in the wells for up to 1 h at coating concentrations of 1 μg/well (data not shown).

The specificity of cell adhesion to Nectin-1 was examined by use of mAbs against Nectin-1 and Nectin-4 to block the interaction between NIH:OVCAR5-N4-over cells and recombinant Nectin-1. Based on the data from the cell adhesion assay (Figure 3A) a length of time for adhesion of 30 min was selected for the inhibition assay, in order to optimize the likelihood of inhibition. A mAb that recognizes the extracellular domain of Nectin-4 inhibited adhesion of the NIH:OVCAR5-N4-over cells by 80% relative to the mouse IgG (immunoglobulin) control mAb, with an IC_50_ value of 0.18 μg/ml antibody (Figure 3B). In addition, we tested two different mAbs against the extracellular domain of Nectin-1 for the ability to block the adhesion of NIH:OVCAR5-N4-over cells, however, these mAbs only inhibited cell adhesion to Nectin-1 by 15% and 35% (Figure 3C).

### Nectin domains involved in cell adhesion

**Figure 4:**
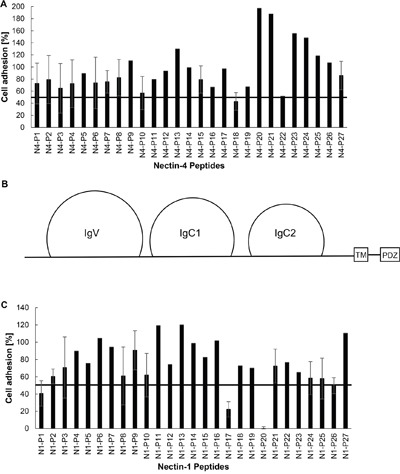
Identification of nectin binding domains Microtiter plates were coated with 0.5 μg/well recombinant human Nectin-1 extracellular domain (R&D Systems). **A.** Triplicate wells were pre-incubated for 30 min with 150 μg/ml Nectin-4 peptides (Table 1). CMFDA stained NIH:OVCAR5-N4-over cells were allowed to adhere in the wells for 30 min. **B.** A diagrammatic representation of the extracellular domain of Nectin-4 and Nectin-1. TM= Transmembrane Domain, PDZ= Afadin binding domain. C. CMFDA stained NIH:OVCAR5-N4-over cells were pre-incubated for 30 min with 150 μg/ml Nectin-1 peptides (Table 1) in triplicate, and then added to the plates for 30 min. Cell adhesion was quantified relative to the adhesion observed with DMSO control; the FU of BSA-coated control wells was subtracted from all samples. The horizontal lines represent 50% adhesion relative to DMSO control. Error bars = SD [%].

**Figure 5:**
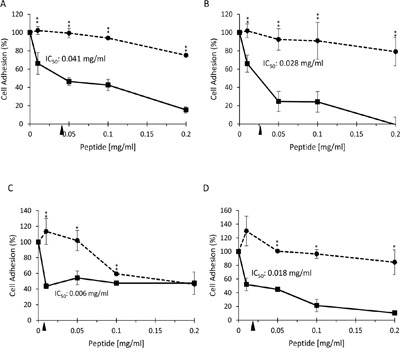
Nectin-4 and Nectin-1 peptides inhibit cell adhesion to recombinant Nectin-1 in a dose-dependent manner Adhesion of CMFDA stained NIH:OVCAR5-N4-over cells to 0.25 μg/ml recombinant human Nectin-1 extracellular domain (Acrobiosystems) was tested after incubation with increasing concentrations of peptides (solid line) compared to the corresponding scrambled control peptide (dashed line) (Table 2). Cell adhesion was quantified relative to the adhesion observed with no peptide added; the FU of BSA-coated control wells was subtracted from all samples. Error bars = SD [%] student's t-test unpaired. **=P<0.01. **A.** N4-P18 and its scrambled peptide. **B.** N4-P22 and its scrambled peptide. **C.** N1-P17 and its scrambled peptide. **D.** N1-P20 and its scrambled peptide. The IC50 value for each active peptide is shown.

Peptides spanning the extracellular domains of Nectin-4 and Nectin-1 (Table 1) were synthesized and screened at 0.15 mg/ml in the cell adhesion assay to identify smaller regions on the nectins that may be involved in cell adhesion. Of the 54 peptides screened, two peptides from Nectin-4 (Figure 4A; peptides N4-P18, and N4-P22) and four peptides from Nectin-1 (Figure 4C; peptides N1-P1, N1-P17, N1-P20, and N1-P26) inhibited adhesion of the NIH:OVCAR5-N4-over cells by ∼50-100% compared to the DMSO control. We synthesized scrambled versions of these six peptides and repeated the cell adhesion assay using increasing concentrations of each peptide and their scrambled control (Table 2). Of the six peptides that were tested, both Nectin-4 peptides and two peptides from Nectin-1 significantly inhibited cell adhesion compared to their scrambled control peptide, with IC_50_ values between 0.006 and 0.041 mg/ml (Figure 5). Three of the peptides inhibited cell adhesion by 90-100%, while peptide N1-P17 blocked cell adhesion by 60% at 0.01 mg/ml. The peptides that inhibited cell adhesion to the greatest extent were derived from the region between the two IgC domains and within IgC2 on both nectins (Figure 4B and Table 2). Several of the Nectin-4 peptides promoted cell adhesion by almost 1.5 fold (N4-P20, N4-P21, N4-P23, N4-P24; see figure 4A).

**Table 1:** Amino acid sequences and domain localization of Nectin-4 and Nectin-1 peptides

Nectin-4 Peptides		Domain	Nectin-1 Peptides	
Name	AA Sequence		Name	AA Sequence
**N4-P1**	PAGELETSDVVTVV	IgV	**N1-P1**	HSQVVQVNDSMYGF
**N4-P2**	VVLGQDAKLPCFYR	IgV	**N1-P2**	GFIGTDVVLHCSFA
**N4-P3**	YRGDSGEQVGQVAW	IgV	**N1-P3**	FANPLPSVKITQVTW
**N4-P4**	AWARVDAGEGAQEL	IgV	**N1-P4**	TWQKSTNGSKQNV
**N4-P5**	ELALLHSKYGLHVS	IgV	**N1-P5**	NVAIYNPSMGVSVL
**N4-P6**	VSPAYEGRVEQPPP	IgV	**N1-P6**	VLAPYRERVEFLRP
**N4-P7**	PPPRNPLDGSVLLR	IgV	**N1-P7**	RPSFTDGTIRLS
**N4-P8**	LRNAVQADEGEYEC	IgV	**N1-P8**	LSRLELEDEGVYIC
**N4-P9**	ECRVSTFPAGSFQA	IgV	**N1-P9**	ICEFATFPTGNRES
**N4-P10**	QARLRLRVLVPPLP	IgV/IgC1	**N1-P10**	ESQLNLTVMAKPTN
**N4-P11**	LPSLNPGPALEEGQ	IgC1	**N1-P11**	TNWIEGTQAVLRAKKGQ
**N4-P12**	GQGLTLAASCTAEG	IgC1	**N1-P12**	GQDDKVLVATCTSANG
**N4-P13**	EGSPAPSVTWDTEV	IgC1	**N1-P13**	NGKPPSVVSWETRL
**N4-P14**	EVKGTTSSRSFKHS	IgC1	**N1-P14**	RLKGEAEYQEIRNP
**N4-P15**	HSRSAAVTSEFHLV	IgC1	**N1-P15**	NPNGTVTVISRYRLV
**N4-P16**	LVPSRSMNGQPLTC	IgC1	**N1-P16**	LVPSREAHQQSLAC
**N4-P17**	TCVVSHPGLLQDQR	IgC1	**N1-P17**	ACIVNYHMDRFKES
**N4-P18**	QRITHILHVSFLAE	IgC1	**N1-P18**	ESLTLNVQYEPE
**N4-P19**	AEASVRGLEDQNLW		**N1-P19**	PEVTIEGFDGNW
**N4-P20**	LWHIGREGAMLKCL	IgC2	**N1-P20**	NWYLQRMDVKLTCK
**N4-P21**	CLSEGQPPPSYNWT	IgC2	**N1-P21**	CKADANPPATEYHWT
**N4-P22**	WTRLDGPLPSGVRV	IgC2	**N1-P22**	WTTLNGSLPKGVEA
**N4-P23**	RVDGDTLGFPPLTT	IgC2	**N1-P23**	EAQNRTLFFKGPINY
**N4-P24**	TTEHSGIYVCHVSN	IgC2	**N1-P24**	NYSLAGTYICEATN
**N4-P25**	SNEFSSRDSQVTVD	IgC2	**N1-P25**	TNPIGTRSGQVEVN
**N4-P26**	VDVLDPQEDSGKQV	IgC2	**N1-P26**	VNITEFPYTPSPPEHGRRA
**N4-P27**	SGKQVDLVSASV	Adjacent to membrane	**N1-P27**	HGRRAGPVPTA

**Table 2:** Amino acid sequences and IC_50_ values of the Nectin-4 and Nectin-1 peptides that inhibited binding of OVCAR5-N4-over cells to recombinant Nectin-1

Nectin-4 Peptides		IC_50_ (mg/ml)	Domain	Scramble AA Sequence
Name	AA Sequence			
**N4-P18**	QRITHILHVSFLAE	0.041	IgC1	HSVLRAHLIIQTEF
**N4-P22**	WTRLDGPLPSGVRV	0.028	IgC2	VLWDPSLTRGVPGR
**Nectin-1 Peptides**		**IC_50_ (mg/ml)**	**Domain**	**Scramble AA Sequence**
**Name**	**AA Sequence**			
**N1-P1**	HSQVVQVNDSMYGF	NA	IgV	VDNQHVMGQSVSYF
**N1-P17**	ACIVNYHMDRFKES	0.006	IgC1	NHSYMCDAIRVFEK
**N1-P20**	NWYLQRMDVKLTCK	0.018	IgC2	DKVYCWLMRLNQTK
**N1-P26**	VNITEFPYTPSPPEHGRRA	NA	IgC2	RPVFEPIEHTPTRNAPGYS

### Nectin-4 expression affects the formation of multicellular spheroids

The formation of tumor spheroids (free floating, multi-cellular aggregates) is a unique feature of ovarian cancer progression. To determine whether the cell adhesion molecule Nectin-4 contributes to spheroid formation, we compared spheroid formation in CAOV3 and NIH:OVCAR5 cells with varying levels of Nectin-4. Single cell suspensions were plated on agarose-coated tissue culture plates and the resulting spheroids were observed over several days to one week.

**Figure 6:**
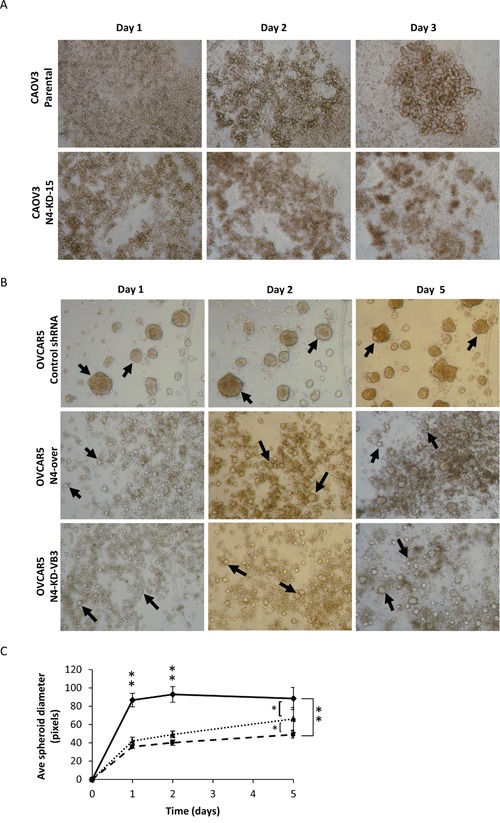
Nectin-4 affects the formation of multicellular spheroids Single cell suspensions of CAOV3 and NIH:OVCAR5 cells were plated in agarose-coated tissue culture plates and spheroids were allowed to form for 5 days. **A.** Representative examples of spheroids formed from CAOV3 parental cells and CAOV3 Nectin-4 knock-down cells (N4-KD-15) after 1, 2, and 3 days in culture, 40X magnification. **B.** Representative examples of spheroids formed from NIH:OVCAR5 control shRNA cells, NIH:OVCAR5 cells that over-express Nectin-4 (NIH:OVCAR5-N4-over), and NIH:OVCAR5 Nectin-4 knock-down cells (NIH:OVCAR5-N4-KD-VB3). Representative examples of spheroids after 1, 2, and 5 days in culture, 40X magnification. Arrows indicate examples of spheroids used for quantification. **C.** Spheroid size was quantified for NIH:OVCAR5 cells expressing a control shRNA (solid line), cells expressing Nectin-4 shRNA (dashed line) or cells that over-express Nectin-4 (dotted line). Error bars = SD. The largest spheroids were quantified (20-90 spheroids/well) in a minimum of 8 wells, for a total of >300 spheroids per cell type. Student's t-test unpaired ** = P<0.01, * = P<0.05.

Within one day CAOV3 cells formed loose aggregates in both the control and Nectin-4 knock-down cell lines (Figure 6A). After 2 days, the CAOV3 control cells formed small, compact, irregularly shaped spheroids, while the CAOV3-N4-KD-15 cells remained loosely aggregated even after 5 days of incubation. It was not possible to quantify the size of the CAOV3 spheroids due to their irregular shape. However, the CAOV3 control cells appeared to coalesce into a single large aggregate after five days, while the CAOV3 Nectin-4 knock-down cells did not (Figure 6A).

NIH:OVCAR5 cells that expressed moderate levels of Nectin-4 (i.e. control shRNA) formed large, round, compact spheroids within 24 h, with very few cells remaining as single cells (Figure 6B). In contrast, the spheroids formed by the NIH:OVCAR5-N4-KD cells were much smaller than the control spheroids, and formed in a background of a large number of single cells or very small cell aggregates (Figure 6B). Surprisingly, spheroid formation in NIH:OVCAR5 cells that over-express Nectin-4 (NIH:OVCAR5-N4-over), was similar to that of the Nectin-4 knock-down cells (Figure 6B). Over the course of 5 days, the size of the spheroids increased for all three cell lines tested; however the NIH:OVCAR5 control shRNA cells (Figure 6C, solid line) were significantly larger in size than the spheroids formed by the NIH:OVCAR5-N4-KD cells (Figure 6C, dashed line) and the spheroids formed by NIH:OVCAR5-N4-over cells (Figure 6C, dotted line).

### Nectin-4 expression alters cell migration

**Figure 7:**
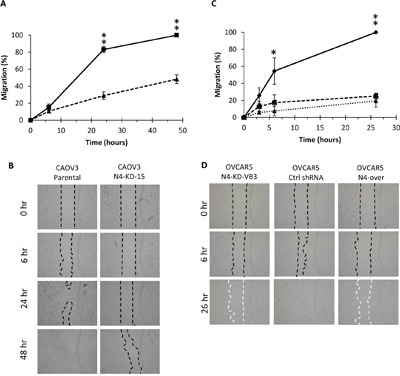
Nectin-4 expression alters cell migration Migration of CAOV3 and NIH:OVCAR5 cells was quantified in a wound healing assay. **A.** Migration of CAOV3 cells was tested and the area of the wound was calculated up to 48 h: CAOV3 parental cells (solid line); CAOV3 N4-KD-15 (dashed line). Error bars = SD. Student's t-test unpaired **=P<0.01. **B.** Representative images of wound healing assay for CAOV3 cells at 0 h, 6 h, 24 h, and 48 h, 40X magnification. Dashed lines indicate the leading edge of the migrating cells. **C.** Migration of NIH:OVCAR5 cells was tested and the area of the wound was calculated up to 26 h: NIH:OVCAR5 control shRNA cells (solid line); Nectin-4 knock-down cells, NIH:OVCAR5-N4-KD-VB3 (dashed line); Nectin-4 over-expressing cells, NIH:OVCAR5-N4-over (dotted line). Error bars = SD. Student's t-test unpaired ** = P<0.01, * = P<0.05. **D.** Representative images of wound healing assay for NIH:OVCAR5 cells at 0 h, 6 h, and 26 h, 40X magnification. Dashed lines indicate the leading edge of the migrating cells, white dashes indicate that some cells migrated into the wound, so that the area was sparsely populated with cells.

Due to the link between Nectin-4 and the actin cytoskeleton [21, 52], we questioned whether Nectin-4 expression could affect ovarian cancer cell migration. To address this, we performed wound healing assays using CAOV3 and NIH:OVCAR5 cells that expressed Nectin-4 or had Nectin-4 expression knocked down by shRNA, and quantified the area of the wounds at several time points. After 24 h, the parental CAOV3 cells expressing Nectin-4 had migrated significantly faster than CAOV3 cells with reduced levels of Nectin-4 expression [CAOV3 N4-KD-15 (Figure 7A-7B)]. After 48 h, the control CAOV3 cells had completely closed the wound, while the Nectin-4 knock-down cells had covered less than 50% of the wound area (Figure 7A-7B).

NIH:OVCAR5 exhibited a significant difference in migration between the NIH:OVCAR5-N4-KD cells and the Nectin-4 control cells after only 6 h (Figure 7C-7D). After 26 h of migration, the NIH:OVCAR5 control shRNA cells had completely closed the area of the wound (Figure 7C-7D), while the NIH:OVCAR5-N4-KD cells had not (Figure 7C-7D). No significant difference in migration rate was observed between the three NIH:OVCAR5-Nectin-4 shRNA clones N4-KD-VB3, N4-KD-VB9, and N4-KD-VB13 (data not shown) that express Nectin-4 in approximately 1% to 13% of the cell population. Similar to the results of the spheroid formation assay, the NIH:OVCAR5-N4-over cells migrated at a rate that was comparable to the Nectin-4 knock-down cells (Figure 7C-7D). In addition, we noted that while the area of the wounds in both the Nectin-4 knock-down cells and the Nectin-4 overexpressing cells was not closed at 26 h, some cells migrated into the wound, so that the area was sparsely populated with cells (see dashed white lines in Figure 7D, bottom panels). These results suggest that Nectin-4 expression affects NIH:OVCAR5 cell migration in a biphasic manner.

### Nectin-4 expression affects cell proliferation

**Figure 8:**
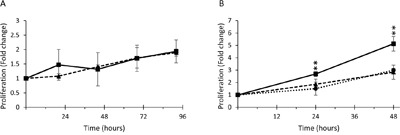
Nectin-4 expression increases cell proliferation in NIH:OVCAR5 cells, but not CAOV3 cells **A.** CAOV3 cells were plated at 15,000 cells per well into 96-well microtiter plates, and grown in OptiMEM® reduced serum media. The number of cells per well was quantified by the CyQUANT® assay at 24 h intervals up to 96 h. Data is represented as the fold change in fluorescence relative to the 2 h time point. No significant difference in cell proliferation was observed between CAOV3 parental (solid line) and Nectin-4 knock-down cells CAOV3-N4-KD-15 (dashed line). Values are an average of two independent experiments (error bars = SD). **B.** NIH:OVCAR5 cells were plated at 8000 cells per well into 96-well microtiter plates and grown in OptiMEM® reduced serum media. The number of cells per well was quantified by the CyQUANT® assay at 24 h and 48 h after plating, and is represented as the fold change in fluorescence relative to the 2 h time point. NIH:OVCAR5 control shRNA cells (solid line); Nectin-4 knock-down cells, NIH:OVCAR5-N4-KD-VB3 (dashed line); Nectin-4 over-expressing cells, NIH:OVCAR5-N4-over (dotted line). Values are an average of 5 independent experiments (error bars = SD). Student's t-test, unpaired: ** p<0.005.

Previous reports have suggested a role for Nectin-4 in cell proliferation [22, 26, 53]. We measured the proliferation rate of CAOV3 and NIH:OVCAR5 cells expressing altered levels of Nectin-4. The CAOV3 cell lines proliferated very slowly, with a doubling time of more than 48 h (Figure 8A). Even after 96 h, neither of the CAOV3 cell lines had proliferated more than ∼2-fold. The CAOV3-N4-KD-15 initially showed a slower proliferation rate, but did not significantly differ from the parental cells (Figure 8A).

In contrast, the NIH:OVCAR5 cells expressing the control shRNA proliferated rapidly, doubling within approximately 18 h, and increasing in number by over 5-fold within 48 h (Figure 8B), while the doubling time for NIH:OVCAR5 cells with Nectin-4 knocked down was over 30 h, and took 48 h to increase in number by 3-fold (Figure 8B). There was no significant difference in proliferation between the three NIH:OVCAR5-Nectin-4 shRNA clones (N4-KD-VB3, N4-KD-VB9, and N4-KD-VB13, data not shown). Similarly, the doubling time for NIH:OVCAR5-N4-over cells was over 30 h, and a 3 fold increase in cell number occurred after 48 h (Figure 8B). Overall, the NIH:OVCAR5 cells that expressed moderate levels of Nectin-4 proliferated significantly faster than either the NIH:OVCAR5 cells that had Nectin-4 knocked down or the NIH:OVCAR5-N4-over cells, again suggesting Nectin-4's biphasic effect.

### Proliferation partially contributes to differences in spheroid formation and cell migration

**Figure 9:**
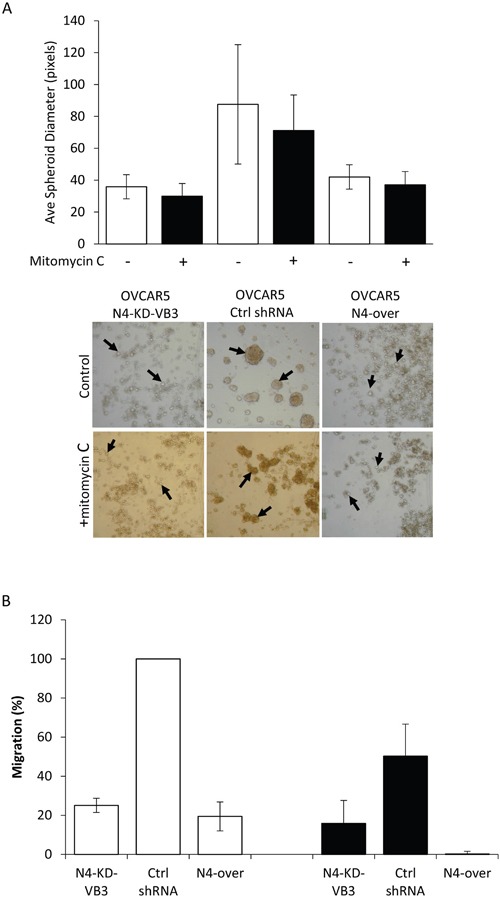
Nectin-4 expression contributes to spheroid formation and cell migration in the absence of cell proliferation NIH:OVCAR5 cells were treated with or without mitomycin C to inhibit cell proliferation and then used in spheroid formation and wound healing assays. **A.** NIH:OVCAR5 cells were treated with or without mitomycin C for 3 h, then single cell suspensions of treated and untreated cells were plated in agarose-coated tissue culture plates and spheroids were allowed to form for 24 h. Top panel: Spheroid size was quantified for NIH:OVCAR5 cells expressing a control shRNA, cells expressing Nectin-4 shRNA (N4-KD-VB3) or cells that over-express Nectin-4 (N4-over) with mitomycin C treatment (black bars) and untreated cells (white bars). Error bars = SD. Bottom panel: Representative examples of spheroids formed from NIH:OVCAR5 control shRNA cells, NIH:OVCAR5 Nectin-4 knock-down cells (NIH:OVCAR5-N4-KD-VB3), and NIH:OVCAR5 cells that over-express Nectin-4 (NIH:OVCAR5-N4-over) with and without mitomycin C treatment. Arrows indicate examples of spheroids used for quantification. **B.** Migration of NIH:OVCAR5 cells was quantified in a wound healing assay in cells treated for 3 h with mitomycin C (black bars) compared to untreated cells (white bars). Area of the wound was calculated after 24 h for NIH:OVCAR5 control shRNA cells, NIH:OVCAR5 Nectin-4 knock-down cells (NIH:OVCAR5-N4-KD-VB3), and NIH:OVCAR5 cells that over-express Nectin-4 (NIH:OVCAR5-N4-over).

In order to determine whether the formation of large spheroids and the rapid rate of migration that we observed for the NIH:OVCAR5 control cells was due to their increased rate of proliferation, we pre-treated the three NIH:OVCAR5 cell lines expressing different levels of Nectin-4 with mitomycin C, a chemical that inhibits cell proliferation by DNA alkylation [54]. Spheroid formation was examined after 24 h with and without mitomycin C treatment. The size of the spheroids formed with and without mitomycin C treatment was essentially unchanged in the NIH:OVCAR5-N4-KD cells and the NIH:OVCAR5-N4-over cells (Figure 9A). The spheroids formed from the NIH:OVCAR5 control cells were somewhat smaller when treated with mitomycin C compared to the untreated cells, however they were still substantially larger than the spheroids formed by the Nectin-4 over-expressing and knock-down cells (Figure 9A).

Migration rates measured in a wound healing assay decreased when the NIH:OVCAR5 cells were treated with mitomycin C. NIH:OVCAR5 control cells treated with mitomycin C migrated to cover only 50% of the exposed area in 24 h, whereas the untreated control cells completely closed the wound (Figure 9B). Mitomycin C treatment also reduced migration in the Nectin-4 knock-down cells N4-KD-VB3, from 25% in the untreated cells to 16% in treated cells (Figure 9B). Treatment with mitomycin C of NIH:OVCAR5 cells that over-express Nectin-4, almost completely inhibited migration (Figure 9B), suggesting that mitomycin C treatment may have adversely affected cell viability, although this was not observed in the spheroid assay, and the cells appeared to be viable and migrating at earlier time points. While the increased cell proliferation in the NIH:OVCAR5 control cells contributes partially to the differences in migration, the alteration in Nectin-4 expression still significantly affects cell migration.

### Nectin-4 expression affects EMT markers

The alterations we observed in cell migration and spheroid formation suggest that Nectin-4 expression may play a role in epithelial to mesenchymal transition (EMT). We tested cell lysates from CAOV3 and NIH:OVCAR5 cells with altered expression of Nectin-4 by Western blot for the expression of four EMT markers: E-cadherin, claudin-1, vimentin, and β-catenin.

**Figure 10:**
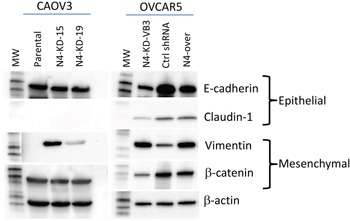
Alterations in Nectin-4 levels affect expression of EMT markers Western blots were performed on cell lysates of CAOV3 and NIH:OVCAR5 cells expressing differing levels of Nectin-4. Blots were probed with antibodies to the epithelial markers E-cadherin and claudin-1, and to the mesenchymal markers vimentin and β-catenin. β-actin was used as a loading control.

In CAOV3 cells, the control and Nectin-4 knock-down cells expressed similar levels of E-cadherin and β-catenin, and none of the cell lines expressed detectable levels of claudin-1 (Figure 10). The CAOV3 control cells did not express the mesenchymal marker vimentin, whereas both of the Nectin-4 knock-down cell lines did (Figure 10). Interestingly, the level of vimentin expression was inversely correlated with the level of Nectin-4 expression [i.e. CAOV3-N4-KD-15, which expresses 25% of parental Nectin-4 (Figure 2B) expressed moderate levels of vimentin, while CAOV3-N4-KD-19, which expresses 70% of parental Nectin-4 (Figure 2B) expressed low levels of vimentin].

NIH:OVCAR5 control cells (which express moderate levels of Nectin-4) have high levels of the epithelial marker E-cadherin, compared to Nectin-4 knock-down cells and N4-over cells (Figure 10). In addition, the epithelial marker claudin-1 is expressed in all three NIH:OVCAR5 cell lines, with higher levels in parental cells and N4-over cells, than the Nectin-4 knock-down cells. The mesenchymal marker vimentin is also expressed in all NIH:OVCAR5 cell lines, however, the control cells have significantly lower vimentin expression than either the Nectin-4 knock-down or overexpressing cells. Another mesenchymal marker, β-catenin, has higher expression in the control and N4-over cells vs. the Nectin-4 knock-down cells (Figure 10). These data suggest that Nectin-4 expression plays a role in EMT (or conversely in MET) in ovarian cancer.

## DISCUSSION

The cell adhesion molecule Nectin-4 is normally expressed in early development and is aberrantly overexpressed in some epithelial cancers, including ovarian cancer [17]. In lung cancer, breast cancer, and pancreatic cancer, expression of Nectin-4 or detection of Nectin-4 in serum was associated with tumors progression or poor survival [22–24, 26]. In this study we showed that both Nectin-4 and its binding partner Nectin-1 are expressed in ovarian cancer primary tumors, ascites cells and omental metastases from patients, as well as in human mesothelial cells, setting the stage for ovarian cancer cell adhesion *in vivo*. In the experiments presented herein, we examined the effect that knocking down Nectin-4 expression in ovarian cancer cell lines has on the various cellular behaviors that underlie metastasis. We found that in addition to playing a role in cell-cell adhesion, knocking down levels of Nectin-4 in the ovarian cancer cells lines CAOV3 and NIH:OVCAR5 affected the formation of multicellular aggregates (i.e. spheroids), and decreased the rates of cell migration, as well as having effects on cell proliferation.

Using an *in vitro* cell adhesion assay, we showed that NIH:OVCAR5-N4-over cells adhere to the extracellular domain of Nectin-1 in a time and dose-dependent matter. However, similar adhesion to the extracellular domain of Nectin-4 was not detected. Although homophilic interaction of nectins has previously been reported [41, 55], the Nectin-4 interaction with itself is weaker than its interaction with Nectin-1 [21]. We showed that mAbs against Nectin-1 and Nectin-4 could inhibit the adhesion of NIH:OVCAR5-N4-over cells to Nectin-1. However, while the mAb against Nectin-4 inhibited cell adhesion dramatically by 80%, neither of the mAbs against Nectin-1 inhibited cell adhesion very robustly in our assay. Fabre et al. [40] have reported that the most distal domains of the nectins (the IgV domains) are sufficient to mediate binding between Nectin-4 and Nectin-1, while the membrane proximal IgC domains affect binding affinity. Reymond et al. [21] also reported that antibodies against the IgV domain of Nectin-1 disrupt the Nectin-4/Nectin-1 interaction. It is possible that the weak inhibition of cell adhesion by mAbs against Nectin-1 is due to the different epitopes recognized by the mAbs. However, as one of the two mAbs (MABT61) that we used to inhibit cell adhesion recognizes the Nectin-1 IgV domain [21], it seems more likely that these mAbs do not recognize the recombinant Nectin-1 that is coated on the plate as well as Nectin-1 on the surface of a cell.

We also screened 54 peptides synthesized from the extracellular domains of Nectin-1 and Nectin-4 for their ability to inhibit cell adhesion to the recombinant human Nectin-1 extracellular domain. Six peptides from Nectin-1 or Nectin-4 inhibited the adhesion of NIH:OVCAR5-N4-over cells to Nectin-1 by at least 50%, and were retested for dose-dependent inhibition of cell adhesion alongside their synthetic scrambled counterparts. Two peptides from Nectin-4 and two peptides from Nectin-1 were able to specifically inhibit cell adhesion by 60-95%. All four of these peptides localized to areas near the IgC2 region, in contrast to the adhesion blocking mAb against Nectin-4 which recognizes the IgV domain [21], and inhibited cell adhesion to a greater extent than the mAbs against Nectin-1. This suggests that the smaller peptides (14-18 amino acids) may have better access to the Nectin-1 protein that is coated on the microtiter plate than the mAbs, or alternatively, that the Nectin-1 mAbs tested are not able to recognize their epitopes outside of the context of the cell surface. Furthermore, it is possible that the peptides binding the IgC domain cause a conformational change, rather than physically blocking the protein-protein interaction. Peptides from the IgV domain that more specifically target the amino acid sequences that have been shown to interact between Nectin-4 and Nectin-1 [56] may better inhibit cell adhesion.

Remarkably, we also observed that several peptides from the IgC2 domain of Nectin-4 promoted cell adhesion to Nectin-1 rather than inhibiting it (Figure 4A), possibly by “bridging” interactions between the Nectin-1 on the plate and Nectin-1 or Nectin-4 on the cell surface. Whether the Nectin-1 or Nectin-4 peptides can inhibit (or promote) other Nectin-4 functions, such as spheroid formation, migration, or proliferation, will be the focus of future studies.

Ovarian cancer is unique in that tumor cells commonly grow within the peritoneal cavity as free floating multi-cellular aggregates (spheroids) in the ascites fluid. We showed that knocking down levels of Nectin-4 expression affected the formation of spheroids in both CAOV3 and NIH:OVCAR5 cells. NIH:OVCAR5 cells expressing Nectin-4 formed significantly larger spheroids than the NIH:OVCAR5-N4-KD cells or NIH:OVCAR5-N4-over cells. Although differing in size, spheroids formed by the three NIH:OVCAR5 cell lines appeared tightly aggregated. This is in contrast to our previous results with the cell adhesion molecule claudin-4, where NIH:OVCAR5 cells depleted of claudin-4 formed spheroids more slowly, and the spheroids formed had increased paracellular permeability and were more loosely aggregated compared to control spheroids [57]. In breast cancer cells, Nectin-4 expression promotes anchorage independent cell survival and proliferation through cell-cell adhesion [50]. A similar mechanism could be working in ovarian cancer patients, as we show in this study that both Nectin-1 and Nectin-4 are expressed in patient ascites cells (Figure 1), which could promote cell aggregation and spheroid formation in cells that have been shed from the primary tumor into the peritoneal cavity. We noted that in both the CAOV3 and NIH:OVCAR5 spheroid assays, very few of the control cells did not assemble into large spheroids, while most of the Nectin-4 knock-down cells were present as single cells or very small multicellular aggregates (Figure 6A-6B). After the initial adhesion between Nectin-4 and Nectin-1, the size of the spheroids may be driven by cell proliferation, as spheroid size is somewhat reduced when proliferation is inhibited by mitomycin C (Figure 9A). Alternatively, the expression of Nectin-4 could be preventing cell death in the control spheroids. Whereas both the large spheroids formed in the NIH:OVCAR5 control cells, as well as the small spheroids and multicellular aggregates formed in the N4-KD cells, appeared viable by trypan blue staining (data not shown) we did not examine the spheroids for markers of apoptosis.

Furthermore, we found that CAOV3 and NIH:OVCAR5 cells expressing Nectin-4 were able to migrate faster than the Nectin-4 knock-down cells in wound healing assays. NIH:OVCAR5 cells expressing a control shRNA completely closed the wound within 24 h. In contrast, the N4-KD and N4-over cells migrated randomly into the wound area and did not completely close the wounds during the 24 h incubation period. This observation suggests that the Nectin-4 knock-down cells lack directional migration or that the cells expressing Nectin-4 may adhere to one another and thus migrate *en masse* instead of migrating as single cells [58, 59]. Takano et al. [26] have shown that COS-7 cells and NIH-3T3 cells that have been genetically engineered to express Nectin-4 have increased migration/invasive potential, as well as increased lamellipodia formation and activated Rac1 signaling. Future studies are needed to determine if this is the mechanism for the increased cell migration in ovarian cancer.

Although the ability of Nectin-4 to promote both the formation of multi-cellular spheroids and cell migration is somewhat antithetical, Sodek et al. [60] have reported that cell lines which form compact spheroids are more invasive than cell lines which form loose aggregates. The formation of compact spheroids is dependent upon actin-myosin contractility [60], which is also the basis for cell motility. In addition, ectodomain shedding of nectins by metalloproteases has been shown to regulate cellular functions such as migration, proliferation, and apoptosis through the binding of the extracellular domain to growth factor receptors [37–39, 42–49]. Lysophosphatidic acid (LPA) stimulates shedding of Nectin-4 [25, 61], and this promotes cell migration in our wound healing assay. LPA has also been shown to promote shedding of E-cadherin, consequently disrupting cell adhesion and promoting motility a ovarian cancer cells [62], supporting the idea that ectodomain shedding of Nectin-4 could regulate cell migration and metastasis in ovarian cancer patients.

In the ovarian cancer cell line NIH:OVCAR5, cells that express Nectin-4 proliferated significantly faster than cells that had Nectin-4 expression knocked down by shRNA or Nectin-4-over cells. A similar proliferative advantage for Nectin-4 expressing cells was recently shown in pancreatic cancer [22] and in lung cancer [26]. In a mouse model, lung cancer cells that express Nectin-4 injected into the flanks of mice showed increased tumor proliferation compared to lung cancer cells without endogenous Nectin-4 expression [26]. However, in our experiments with CAOV3 cells there was no increase in proliferation associated with Nectin-4 expression, suggesting that different cell lines may respond differently to Nectin-4 expression depending upon which signaling pathways are active. Although our experiments showed reduced proliferation in NIH:OVCAR5-N4-KD cells, we cannot rule out the possibility that the proliferation rate is not different from the parental cells, but rather that the N4-KD cells are undergoing apoptosis.

One surprising result of our experiments using NIH:OVCAR5 cells was the phenotype of the N4-over cells with respect to cell migration, proliferation, and spheroid formation. Since the N4-KD cells exhibited decreased migration and proliferation and smaller spheroid formation compared to control cells, we anticipated that the N4-over cells might have increased migration and proliferation compared to the control cells. Instead, the N4-over cells behaved similarly to the N4-KD cells in our functional assays. This result suggests that the overexpression of Nectin-4 may be disrupting complexes formed between Nectin-4 and other proteins important for its function, such as afadin [21, 63], or perhaps, in the case of cell migration, that the additional adhesive strength provided by the Nectin-4 overexpression causes the cells to migrate more slowly. Whether the expression levels of Nectin-4 in ovarian cancer patients affects survival or response to therapy is not known.

We also examined protein expression of several EMT markers in both CAOV3 and NIH:OVCAR5 cells with varying amounts of Nectin-4. In CAOV3 cells, N4-KD cells expressed higher levels of the mesenchymal marker vimentin than the control cells. Expression of vimentin has been associated with invasive behavior in both ovarian cancer cell lines and patient ascites [13]. In NIH:OVCAR5 cells, both parental and N4-over cells had increased amounts of E-Cadherin, β-catenin, and claudin-1, compared to the knock-down cells, suggesting Nectin-4 plays a role in EMT and ovarian cancer progression. Increased expression of claudin-1 has been associated with a more aggressive phenotype in breast cancer [64], and is associated with poor survival in ovarian cancer [65]. While somewhat contradictory to claudin-1 as a tumor suppressor, recent studies suggest that higher expression levels of claudin-1 induce EMT in human liver cells through the activation of the ERK signaling pathway [66]. Our own unpublished results indicate that NIH:OVCAR5-N4-over cells have upregulated levels of ERK signaling compared to the parental cells, but further studies are needed to assess the impact of Nectin-4 on cell signaling.

In this study, we have shown that the cell adhesion molecule Nectin-4 promotes cell adhesion and migration, as well as proliferation and the formation of multicellular spheroids. We have also shown that Nectin-4 and its binding partner Nectin-1 are expressed in ascites cells, primary tumors, and omental metastases from ovarian cancer patients, as well as human mesothelial cells. Taken together, these data suggest that Nectin-4 may play a role in local ovarian cancer progression by promoting tumor proliferation, migration, and invasion into the mesothelial cell lining of the peritoneum. However, a new study has demonstrated that ovarian cancer may also spread via hematogenous metastasis [3]. In pancreatic cancer, Nectin-4 expression detected by immunohistochemistry correlated with vascular endothelial growth factor expression by quantitative RT-PCR [22]. High Nectin-4 expression was also associated with increased microvessel density, suggesting a role for Nectin-4 in angiogenesis [22] and potentially in hematogenous metastasis as well.

The expression of Nectin-4 on the surface of ovarian cancer tumors [17–19] suggests that Nectin-4 is a valid target for therapy, and our *in vitro* data showed that a mAb against the IgV domain of Nectin-4 almost completely blocked ovarian cancer cell adhesion to Nectin-1. Pavlova et al. [50] used this same mAb in a mouse xenograft model of breast cancer and observed disruption of tumor cell adhesion and reduced tumor growth *in vivo* compared to tumors treated with control IgG [50]. In contrast, the mAb which Takano et al. generated against the extracellular domain of Nectin-4 [26] was not tested for its ability to block cell adhesion. In their studies, treatment with their mAb against Nectin-4 did not reduce tumor size in lung cancer xenografts [26]; indicating that blocking Nectin-4 cell adhesion may be an important component of therapeutic efficacy for a mAb in this setting.

Recently, Challita-Eid et al. [27] reported the development of an antibody-drug conjugate targeting Nectin-4 that is in preclinical development. The antibody recognizes an epitope in the IgV domain, and is able to block the interaction of Nectin-1 and Nectin-4 on the surface of rat fibroblast cells. The antibody-drug conjugate induced dose-dependent cytotoxicity *in vitro*, and significantly reduced growth or caused tumor regression in mouse xenograft models of human breast, bladder, pancreatic and lung cancers [27]. This antibody-drug conjugate is currently in clinical trials for patients with solid tumors that express Nectin-4 [28]. Nevertheless, understanding the biology of Nectin-4 in ovarian cancer progression is still critical to facilitate its development as a therapeutic target.

## MATERIALS AND METHODS

### Cell culture

The human ovarian cancer cell lines NIH:OVCAR5 (Judah Folkman, Harvard University) [67] and CAOV3 (Robert Bast, MD Anderson) were received in 1995 and viably stored in liquid nitrogen. After thawing, cells were grown in complete medium [RPMI 1640 media containing 10% fetal bovine serum (FBS) for NIH:OVCAR5 cells or DMEM + 10% FBS for CAOV3] at 37°C in a humidified incubator with 5% CO_2_.

### Antibodies

Mouse monoclonal antibodies (mAb) against human Nectin-4 were purchased from Millipore (Billerica, MA) (MABT64, clone N4.61) and R&D Systems (Minneapolis, MN) (MAB2659, clone #337516). Mouse mAbs against human Nectin-1 (MAB2880, clone #610835) was purchased from R&D Systems and Millipore (MABT61, clone R1.302). Negative control antibodies included a mouse mAb IgG_2b_ (MAB0041, Clone #133303, R&D Systems), a mouse mAb IgG_2a_ (MAB003, Clone #20102, R&D Systems), and a polyclonal mouse IgG (ab37355; Abcam, Cambridge, MA). EMT antibodies against Vimentin (D21H3), Claudin-1 (D5H1D), β-Catenin (D10A8), and E-Cadherin (24E10) were from Cell Signaling Technology (Danvers, MA). A mAb against β-actin (clone AC-74, Sigma-Aldrich, St. Louis, MO) was used as a loading control for Western blots.

### Flow cytometry

Confluent monolayers of cells were detached from 75 cm^2^ tissue culture flasks using Accutase cell dissociation buffer (Innovative Cell Technologies, San Diego, CA), washed, and labeled with mouse IgG, a mAb against Nectin-4 (MAB2659, clone #337516) or a mAb against Nectin-1 (MAB2880, clone #610835) using 2.5 μg mAb/10^6^ cells in Flow Buffer [phosphate buffered saline (PBS) containing 2.5% newborn calf serum and 0.02% sodium azide] for 30 min at 4°C [18]. Cells were washed and incubated with goat anti-mouse IgG F(ab’)2 (Jackson ImmunoResearch, West Grove, PA), then washed again and incubated with streptavidin-allophycocyanin conjugate (Jackson ImmunoResearch, West Grove, PA) for 30 min each. Cells were washed and fixed in Flow Buffer containing 1% formaldehyde and analyzed in the University of Minnesota Flow Cytometry Resource with a BD Accuri™ C6 flow cytometer (BD Biosciences, San Jose, CA) using the Accuri™ software.

### Reverse Transcription – Polymerase Chain Reaction (RT-PCR)

Total cellular RNA was isolated from cell lines or snap frozen patient tissues using the RNeasy Mini kit (Qiagen; Hilden, Germany) per manufacturer's instructions. 50-100 ng of total RNA was amplified using the Access RT-PCR System (Promega Corporation, Madison, WI), as previously described [18]. Primers: Nectin-4 Forward (5′- CAAAATCTGTGGCACATTGG -3′) and Reverse (5′- GCTGACATGGCAGACGTAGA -3′), GAPDH Control Forward (5′- ACCACAGTCCATGCCATCAC -3′) and Reverse (5′- TCCACCACCCTGTTGCTGTA -3′), Nectin-1 Forward (5′- GGACCACGCTAAATGGCTCT -3′) and Reverse (5′- GGGGTGTAGGGGAATTCTGTG- 3′), β-actin Forward (5′- GGCCACGGCTGCTTC-3′) and Reverse (5′- GTTGGCGTACAGGTCTTTGC-3′). Amplification products were visualized on a 1% agarose gel stained with SYBR^®^ Gold (Invitrogen™).

### Overexpression of Nectin-4

A cDNA of the full length Nectin-4 isoform cloned in the p3XFLAG-myc-CMV-25 expression vector (Sigma-Aldrich) was kindly provided by Dr. Marc Lopez (Centre de Recherché en Cancérologie de Marseille, Marseille, France). NIH:OVCAR5 cells were grown to 70% confluency and transfected using Lipofectamine^®^ LTX and Plus™ Reagent (Invitrogen™, Grand Island, NY). Cells were subsequently selected using G418/Geneticin^®^ (Invitrogen™). Overexpression of Nectin-4 was verified by flow cytometry, and fluorescence activated cell sorting (FACS) was used to select for cells that expressed high levels of Nectin-4 compared to the parental cell line. The NIH:OVCAR5 cell line that was generated, which stably overexpressed Nectin-4, will be hereafter referred to as NIH:OVCAR5-N4-over.

### Lentiviral transduction

shRNA lentiviral particles targeting human Nectin-4 or control shRNA lentiviral particles were purchased from Santa Cruz Biotechnology, Inc. (Dallas, TX) and used to transduce cells according to the manufacturer's instructions. NIH:OVCAR5 cells were transduced with 10-20 μl of high titer lentiviral particles in 2 μg/ml Polybrene® (hexadimethrine bromide, Sigma-Aldrich) in complete medium. CAOV3 cells were transduced with 10 μl of lentiviral particles in 8 μg/ml Polybrene®. Infected cells were selected with puromycin and single cell clones were derived by limiting dilution. Clones with reduced expression of Nectin-4 were detected by RT-PCR and verified by flow cytometry.

### Synthetic peptides

Twenty-seven peptides of 14 amino acids in length were designed from the extracellular domain of Nectin-4 so that the sequences overlapped by two amino acids (Table 1). The Nectin-1 peptides synthesized ranged from 11 to 17 amino acids in length; and were selected to correspond to similar regions in Nectin-4 (Table 1). Scrambled versions of the six peptides that caused the greatest inhibition of cell adhesion to Nectin-1 were also synthesized (Table 2). All peptides were synthesized with a free amino terminus and an amide at the carboxy terminus (CONH2). The peptides were synthesized by Aapptec (Louisville, KY) where they were purified by high pressure chromatography, and the sequences were verified by mass spectrometry to be >95% pure. Peptides were dissolved in dimethyl sulfoxide (DMSO; Sigma-Aldrich) at a concentration of 50 mg/ml, aliquoted, and stored at -80°C.

### Cell adhesion

Black, clear-bottom 96-well microtiter plates (Corning, Imported Costar, Corning, NY) were coated with various concentrations of recombinant human Nectin-1 extracellular domain (R&D Systems or Acrobiosystems, Newark, DE), recombinant human Nectin-4 extracellular domain (R&D Systems), or bovine serum albumin (BSA; Probumin Diagnostic Grade, MilliPore) diluted in PBS. Covered plates were incubated overnight at 37°C in a humidified incubator. After washing with PBS plus Ca^2+^/Mg^2+^ containing 2 mg/ml ovalbumin (Sigma-Aldrich), the plates were blocked for 1 h at 37°C with 1% BSA in PBS without Ca^2+^/Mg^2+^. Confluent monolayers of NIH:OVCAR5 cells or NIH:OVCAR5-N4-over cells were detached with Accutase, and then filtered through a 40 μm cell strainer (BD Biosciences, San Jose, CA) to remove cell aggregates. The single cells were stained with CellTracker Green CMFDA (5-chloromethylfluorescein diacetate; Invitrogen™) according to the manufacturer's protocol. After washing the plate, 30,000 cells were added to each well in 50 μl of RPMI 1640 media containing 2 mg/ml ovalbumin and allowed to adhere for 15-60 min at 37°C. Plates were vigorously washed four times and read in a BioTek Synergy fluorescent microplate reader (BioTek, Winooski, VA). Experiments were conducted in triplicate.

### Inhibition of cell adhesion with mAbs

Microtiter plates coated with 0.25 μg/well recombinant human Nectin-1 (Acrobiosystems) as described above were pre-incubated for 30 min at room temperature with mAbs against Nectin-1 or mouse IgG diluted in PBS at 0.25 to 3 μg/ml. CMFDA-stained NIH:OVCAR5-N4-over cells were then added to the wells and cell adhesion was quantified after a 30 min incubation as described above. In parallel studies, CMFDA-stained NIH:OVCAR5-N4-over cells suspended in RPMI 1640 with 2 mg/ml ovalbumin were pre-incubated for 30 min at room temperature in 0.25 to 3 μg/ml of the mAb against Nectin-4 or mouse IgG, and the cell-mAb suspension was subsequently added to the 96-well plate and incubated for 30 min. Experiments were conducted in triplicate and repeated three times.

### Definition of nectin cell adhesion domains by synthetic peptides

Microtiter plates coated with 0.5 μg/well recombinant human Nectin-1 extracellular domain (R&D Systems) as described above were pre-incubated for 30 min at room temperature with 150 μg/ml Nectin-4 peptides in PBS in triplicate. CMFDA-stained NIH:OVCAR5-N4-over cells were then added to the wells and cell adhesion was quantified after a 30 min incubation as described above. In parallel studies, CMFDA-stained NIH:OVCAR5-N4-over cells were pre-incubated for 30 min at room temperature with 150 μg/ml Nectin-1 peptides in PBS in triplicate, and then the cells and peptides were added to the 96-well plate followed by a 30 min incubation. In each experiment, DMSO, which had been used to reconstitute the desiccated peptides, was used at a comparable dilution as a control. Peptides were screened at 150 μg/ml, and those selected for effective inhibition of cell adhesion were analyzed for dose-dependent inhibition alongside their scrambled counterparts. Dose curves were conducted as described above, except the microtiter plates were coated with 0.25 μg/well recombinant human Nectin-1 extracellular domain (Acrobiosystems), and the peptides and scrambled controls were diluted from 0.01-200 μg/ml.

### Spheroid formation

Spheroids were formed from the cell lines described above using the liquid overlay method [8, 9]. One milliliter of a single cell suspension was seeded at a concentration of 50,000 cells/ml in complete media, into 24-well tissue culture plates coated with 0.5% Seakem agarose (in complete media). Plates were incubated at 37°C in a humidified incubator with 5% CO_2_. At various time points up to 5 days, the plates were removed from the incubator and the spheroids were photographed with a Nikon CoolPix digital camera mounted on an Olympus CK2 inverted microscope with a 4X objective. The size of the spheroids was determined using the measure tool in Adobe Photoshop. The largest spheroids were quantified (20-90 spheroids/well) in a minimum of 8 wells, for a total of >300 spheroids per cell type.

### Cell migration

Cells were diluted to a concentration of 700,000-10^6^ cells/ml in complete media, and 70 μl was plated into each chamber (0.22 cm^2^ growth area) of an ibidi_®_ wound-healing culture-insert (ibidi_®_ Verona, WI) in triplicate in a 100 × 20 mm dish (Corning, Falcon, Corning NY). Cells were grown overnight at 37°C in a humidified incubator with 5% CO_2_ or until the chambers were confluent, prior to removal of the culture insert. Migrating cells were incubated in OptiMEM^®^ containing 10 μM lysophosphatidic acid (LPA Cayman Chemical, Ann Arbor, MI) and photographed initially (t = 0 h), and then up to 48 h with a 4X objective. Image analysis was performed using ImageJ software [68] with the Scratch Assay Analyzer from the MiToBo plug in. Three independent experiments were performed on each cell line.

### Cell proliferation

Cell proliferation was quantified using the CyQUANT® assay (Invitrogen™) according to the manufacturer's instructions. Briefly, 8,000-15,000 cells were seeded per well into 96-well microtiter plates in complete media and allowed to adhere for 2 h at 37°C. After 2 h, the media was replaced with OptiMEM^®^ reduced serum media and the cells were grown at 37°C. Cells were collected at 24 h intervals for up to 96 h. At each time point, the media was removed, plates were frozen to lyse cells, and the fluorescence was measured in a BioTek Synergy fluorescent microplate reader after adding the CyQUANT^®^ GR dye. Proliferation was calculated as the fold change in fluorescence relative to the 2 h time point. Experiments were repeated 2-5 times in quadruplicate.

### Mitomycin C treatment

Mitomycin C (Sigma-Aldrich, M287) was reconstituted at 0.4 mg/ml in PBS, pH 7.4, according to the supplier's instructions. Prior to spheroid assays, adherent NIH:OVCAR5 cells were pretreated for 3 hours at 37°C, in a tissue culture flask 10 μg/ml mitomycin C diluted in complete RPMI media. Flasks were rinsed with PBS and cells were detached as described for the spheroid assay. For migration assays, cells were plated in ibidi_®_ wound-healing culture-insert incubated overnight, then the media was removed from the culture inserts and replaced with 25 μg/ml mitomycin C in complete RPMI. After 3 hours at 37°C, the culture inserts were removed and the cells were washed with PBS and cell migration was measured as described above.

### Western blots

Total protein was extracted from cell monolayers using the Blue Loading Buffer Pack (Cell Signaling Technology) according to the manufacturer's protocol. Equal volumes of each extract were loaded onto a Mini-PROTEAN®-TGX ™ 4-20% precast gel (Bio-Rad, Hercules, CA) and transferred to PVDF (Polyvinylidine Diflouride, GE Healthcare Life Sciences, Pittsburgh, PA). EMT marker expression was probed using antibodies from the CST Epithelial-Mesenchymal Transition Antibody Sampler Kit, and protein size was estimated with the Biotinylated Protein Ladder Detection Pack (Cell Signaling Technology) according to the manufacturer's instructions. Chemiluminescence was detected with SuperSignal West Femto substrate (Thermo Scientific, Waltham, MA) and documented with a FluorChem E system (ProteinSimple, San Jose, CA).
